# Editorial: Improving decoding of neuroinformation: towards the diversity of neural engineering applications

**DOI:** 10.3389/fnhum.2023.1270696

**Published:** 2023-08-29

**Authors:** Luz María Alonso-Valerdi

**Affiliations:** Tecnologico de Monterrey, Escuela de Ingeniería y Ciencias, Monterrey, NL, Mexico

**Keywords:** neural activity, neurotechnology, neural interfaces, neuroengineering, human-environment interaction, brain-machine interfaces

This Research Topic explores creative neural engineering solutions to improve current human-environment and human-machine interactions in different contexts, including clinical, social, cognitive, environmental, applicative, and psychophysiological fields. The responses to the call tended to consider cognitive and applicative approaches. On one hand, cognitive research currently requires (1) more creative and realistic methods, where ecological stimuli could be feasible; (2) integrative models supported by a wide range of disciplines such as neurosciences, computational sciences, electronic engineering, behavioral and cognitive sciences, and many others; and (3) proposals that promote diversity. On the other hand, applicative research concerning human-machine interaction is searching for new solutions which include the user as an element of the system structure. In this respect, the guidance of the user system toward system control is crucial for the appropriate functioning of the whole system. Such guidance is achieved through constant and accurate feedback being provided during human-machine interaction to achieve the highest possible performance.

The Research Topic includes four articles outlining scientific advances in diverse neural engineering applications toward improving the decoding of neuro-information.

The first article by Duville et al. undertook a behavioral and neurophysiological approach to the emotional perception of human and naturalness-reduced synthesized versions of emotional and neutral utterances. The authors found that processing emotional prosodies still preserved when listening to less natural voices (Duville et al.). This finding is of high relevance in the development of smart technology, particularly for voice assistants. To date, the optimization of the persuasive capabilities of voice assistants has been challenging since contradictory solutions have been proposed (Herder and Herden, 2023). The authors compared the effect of the empathic and authoritative phrasing of persuasive recommendations. However, no success was achieved owing to individual differences and user contexts. Therefore, synthesized voices could be helpful for persuasive voice assistants since emotional processing is preserved but with simpler language processing.

Second, Agoalikum et al. investigated the structural differences among attentional deficit and hyperactivity disorder (ADHD) in infant, adolescent, and adult patients. The study aimed to find the causal relationship between the right pallidum and the rest of the brain. They demonstrated the effective connectivity of the right pallidum and the pathophysiology of ADHD (Agoalikum et al.). The palladium has not only been associated with motor functions but also with motivation and reward (Smith et al., 2009). The results of Agoalikum et al. provide insight into the brain function of ADHD, which in turn expands knowledge of neurodiversity. This concept refers to differences in individual brain function and the behavioral traits of dyspraxia, dyslexia, ADHD, dyscalculia, the autistic spectrum, and Tourette syndrome, which are regarded as part of the normal variation in the population. Knowing more about how the brain functions in a neurodiverse population could help create learning tools for teachers, helping them to develop classes and content for their neurodiverse students. Many presently used teaching and assessment approaches are ambivalent and inflexible, and staff have a low awareness of the needs of neurodiverse students (Clouder et al., 2020).

In the third contribution, Savić et al. propose a brain-computer interface (BCI) system based on electro-tactile control. The authors achieved an improvement from 75.1 to 88.1% for all participants in the study (Savić et al.). Previously, it had been demonstrated that electro-tactile stimulation is very effective for human messaging comprehension and it can even substitute verbal information transmission (Kim et al., 2004). This prototype proposal promotes the multisensory control of BCI systems. The higher the enriched sensory environment, the higher the perception of the environment obtained. Interaction with the environment is considered more human-like when the information is transmitted through diverse modalities that can be perceived through all the senses (Shivappa et al., 2010).

Finally, the study by Ivanov and Chau outlines two trial-wise adaptations (sliding window and weighted average) of Riemannian geometry-based user-performance metrics, provided as feedback after each individual trial increases classifier performance, and compares it to a conventional classification output (Ivanov and Chau). Feedback is when information is provided to a learner concerning their understanding of a task, helping students to master such task. To achieve this purpose, feedback must be heard, understandable, and actionable (Mandouit and Hattie, 2023). The project presented in this Research Topic meets these three requirements. The study aimed to reduce BCI inefficiency, using different modes of feedback and examining how they promote learning, motivation, and emotions. As Mandouit and Hattie (2023) state, feedback must answer four questions, asking (1) what is done well; (2) where can improvement be made; (3) how can they improve; and (4) what should they do next time?

As shown in the studies included in this Research Topic, to improve the decoding of neuro-information and achieve more feasible and effective neural engineering applications, it is necessary first to understand how the human brain works, and then, how we can use and improve this functionality. If neural engineers make an effort to provide deeper insights into human brain functionality, the application and efficiency of our systems are going to be more realistic and higher. Duville et al. could have extended the implications of their study by illustrating technological development benefits. Agoalikum et al. might have concrete applications of their findings in neurodiverse learning, particularly in ADHD populations. Savić et al. could further justify the context of using tactile-based control in terms of human reaction time and environmental perception. Finally, Ivanov and Chau could have enriched their discussion by highlighting the importance of feedback in learning processes to improve BCI user performance. Although the human brain is only 2% of body mass and accounts for 20% of body oxygen consumption, it determines human learning and human adaptation. Once neural engineers move toward understanding more about functionality, neurotechnology will advance even further (Figure 1).

**Figure 1:**
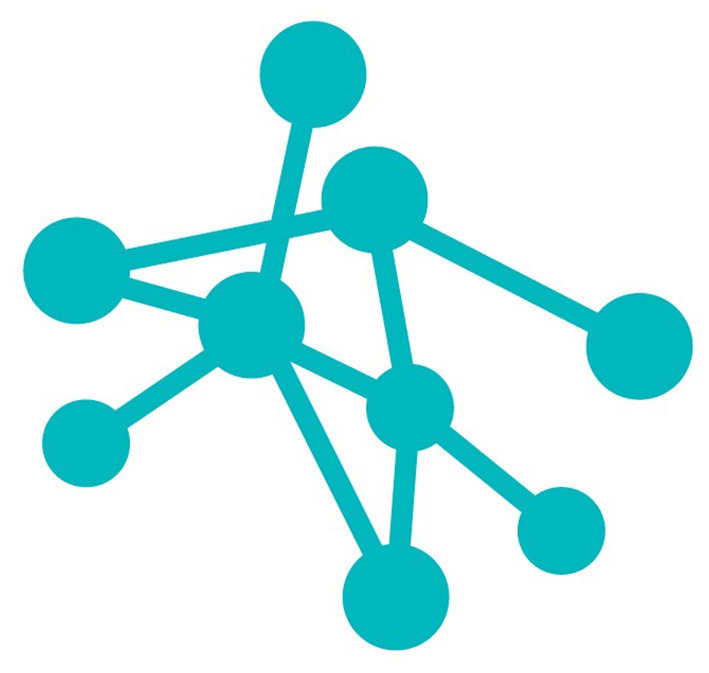
One of the major challenges in neurotechnological advances is understanding the neuronal communication. A way of illustration is the recent creation of spiking neural networks based on the neuron functioning.

## Author contributions

LMA-V: Conceptualization, Investigation, Project administration, Writing—original draft, Writing—review and editing.
